# Redefining the design innovation process: Embedding sustainability through AI-generated camping cookware

**DOI:** 10.1371/journal.pone.0355687

**Published:** 2026-08-17

**Authors:** Guanfang Weng, Chunlei Xu, Jeremy Cenci, Dongmei Peng, Mingjie Shen, Jiazhen Zhang

**Affiliations:** 1 College of Industrial Design, Shandong University of Art and Design, Jinan, Shandong, China; 2 Wales College, Lanzhou University, Lanzhou, Gansu, China; 3 Faculty of Architecture and Urban Planning, University of Mons, Mons, Belgium; Industrial University of Ho Chi Minh City, VIET NAM

## Abstract

With the increasing interest in glamping, the demand for more functional camping cookware has grown, driving innovation in its design. This study aims to explore innovative solutions for camping cookware, and establishes a systematic product conceptual design process through the integration of artificial intelligence-generated content (AIGC) and appropriate data analysis tools. Following the case study, by integrating sustainability-related principles, the study proposes an AIGC-assisted design process for sustainable product design, with its comparative effectiveness yet to be validated. Specifically, combining the GIOIA methodology with the Delphi method and Fuzzy Analytic Hierarchy Process (FAHP) to systematically identify and structure user expectations. These insights informed the development of a comprehensive requirements model for cookware design. Guided by this model, creative concepts were produced using generative tools such as Midjourney and Stable Diffusion, constrained by structured prompt cards. The resulting design outputs were assessed using VIKOR and Fuzzy Comprehensive Evaluation (FCE) to evaluate the prioritization of design alternatives and users’ satisfaction with the resulting concept.The findings indicate that functional requirements and safety requirements are the primary considerations for camping cookware, especially usability and Ergonomic adaptability. In comparison, emotional requirements such as cultural resonance, personalized customization, and aesthetic appeal are not key focuses. In addition, AIGC can accurately reproduce design schemes, improve design efficiency and promote creativity generation.. The proposed framework demonstrates potential for aligning digital creativity with sustainability goals in consumer product design.

## 1. Introduction

Glamping, a blend of “glamour” and “camping,” was first introduced in Europe and the United States [1]. Unlike traditional camping, glamping combines functionality and emotional needs through carefully designed scenarios, personalized services, and diverse experiences, offering users a comfortable, immersive outdoor lifestyle. The global luxury camping market, valued at USD 3,205 million in 2024, is projected to grow at a CAGR of 10.9% over the next five years [2].

The rise of glamping has driven innovation in camping products, elevating expectations beyond basic functionality to include user-centered design and information technology integration. These advancements provide lightweight solutions that enhance the glamping experience. Camping cookware, as a key component, facilitates collective cooking and strengthens the emotional and collaborative bonds between campers. However, most intelligent camping cookware remains in the conceptual stage, focusing more on utility than on creating engaging, user-centered experiences. Current products mainly meet basic cooking needs and lack intelligent interaction, multi-modal synergy [3], and consideration of user experience [4], falling short of the glamour associated with modern camping trends [5]. As lifestyle quality improves and personalized demands grow, users now seek more than just functionality—they desire intelligent experiences and fulfillment of latent needs. Therefore, improving the innovative design of camping cookware is crucial to provide a personalized glamping experience, integrating innovative technologies to create a high-quality, multi-level system.

In recent years, the rapid advancement of artificial intelligence has led to a revolution in design, with AIGC technology simulating the human creative process to autonomously generate artworks in various forms, including audio, text, and images [6]. This technology not only provides designers with innovative tools but also opens new possibilities for product innovation and sustainability [7].Through data-driven capabilities, AIGC enables automated and augmented innovations that enhance design efficiency and inspire creative expression and paradigm shifts [8]. However, the content it generates may lack originality and human aesthetic judgment, limiting its ability to solve complex creative design challenges independently. Blind application of AIGC can lead to suboptimal results or even counterproductive outcomes. In this context, the designer’s decision-making role becomes increasingly critical, with human-AIGC collaboration offering a model that leverages the strengths of both. This collaboration represents a new paradigm in innovation, combining human decision-making with intelligent algorithms and integrating technical indicators with value objectives [9].

The design community faces the dual challenge of fostering innovation while addressing sustainability concerns in modern society. On one hand, camping cookware design must evolve to meet changing user needs. On the other, in the era of sustainable development, there is increased emphasis on minimizing environmental impact throughout a product’s life cycle. Additionally, with the rise of AIGC technology, designers are compelled to transcend traditional design limits and seek new sources of inspiration and methods. This raises the need for more effective human-AIGC collaboration, making it crucial to find a suitable path that bridges the gap between user needs, innovation, and sustainability. In this paper, we take camping cookware as an entry point to explore the following issues in depth:

Q1: What are the cookware design requirements needed by users in the context of glamping? How to use AIGC technology to accurately reproduce these characteristics?

Q2: What is AIGC’s human-machine collaborative product design process?

Q3: What will the product innovation design process look like in the future when AIGC is integrated with sustainable design principles?

This study aims to explore user needs for camping cookware within the context of glamping and to propose novel conceptual design solutions. Based on a case study, a design process integrating AIGC with user needs is developed. To better align with broader environmental concerns, sustainability-related principles are incorporated into the design process, and a conceptual framework for a sustainable product design process is proposed, providing a basis for future empirical research. The proposed approach may contribute to the evolution of design practice and offer new insights into the development of design theory. Furthermore, this study encourages the design field to move beyond conventional methods, actively engage with emerging technologies, and integrate advanced design approaches in response to societal needs, thereby supporting the sustainable development of product design.

## 2. Literature review

### 2.1. Sustainable and innovative design of camping cookware

Glamping, a popular leisure activity among Generation Z, continues to gain momentum due to its short-distance, lightweight, and multimodal features [10]. It caters to the contemporary urban youth’s demand for short-distance travel and weekend micro-vacations, while also driving innovation in outdoor products. The innovative design of these products has drawn attention from design scholars. Milohnić I, using data from camping enthusiasts, camp managers, and product developers, explored innovative mobile home designs, highlighting the significance of accommodation equipment in enhancing the camping experience [11]. Wang L examined the importance of outdoor clothing attributes and consumer decision-making in response to the growing interest in sustainable apparel [12]. Buckley R proposed a psychological tourism framework to guide product innovation aimed at enhancing travel well-being [13]. Despite these contributions, research on camping product design, particularly cookware in the context of glamping, remains limited. Most studies focus on theoretical aspects, lacking practical research cases and innovative perspectives across the entire product design process.

Sustainable design stems from the concept of sustainable development, reflecting the design community’s response to the relationship between human development and environmental issues, and the ongoing process of seeking change [14]. While the definition of “sustainable design” remains debated in academic circles, it is closely related to “green design,” “eco-design,” “low-carbon design,” and “environmental design” [15]. Unlike traditional design, which focuses on material products, sustainable design integrates “products and services” to create “sustainable solutions” that meet consumer needs. This approach prioritizes “results” and “benefits” over material consumption, aiming to reduce resource waste and environmental pollution, while improving the quality of life in society.

In product design, sustainable design theory is receiving increasing attention from designers and manufacturers. The literature review shows that the theoretical framework of sustainable design contains several key aspects. First, sustainable design prioritizes the selection and use of product materials. L.Y. emphasizes the importance of using environmentally friendly, renewable or recyclable materials in furniture manufacturing as a key approach to sustainability [16]. Second, it focuses on product recycling. Kim and Moon demonstrate how a sustainable electric shaver product family can reduce environmental impacts while meeting customer needs and maintaining profitability through modular manufacturing [17]. Hossain et al. propose the Sustainable Modular Product Architecture (SMPA), which enhances assembly ease and recyclability, thereby reducing complexity and waste [18]. Finally, sustainable design stresses product life-cycle management. Yang D. et al. argue that designers should consider maintainability, disassembly, and recyclability to extend product lifespan and minimize waste [19]. Schöggl J.P. introduces a new framework for sustainable product development (CSPD), allowing qualitative assessment of environmental, economic, and social aspects early in the product development process, considering the entire product life cycle [20].

Overall, sustainable design theory provides the necessary guiding principles and methods for product design to help designers and manufacturers strike a balance between aesthetics, functionality, and environmental considerations. According to the aforementioned literature, sustainable product design focuses on quality material selection, improving product durability, and reducing environmental impact. Researchers have rarely explored sustainable product design processes, especially the human-machine collaborative sustainable design process integrated with AIGC. This study emphasizes sustainable innovation for camping cookware, focusing on the exploration of user needs. At the same time, it emphasizes the innovation of sustainable product design process and the need for human-AIGC collaborative design process, and this study is expected to provide new insights and innovative ideas for sustainable development in the field of product design.

### 2.2. AICG technology overview

Generative Artificial Intelligence enables the generation of text, images, and videos by integrating various data, algorithms, and models [21]. With advancements in technology and the continuous iteration of algorithms—especially the development of generative adversarial networks and diffusion models—AIGC has rapidly progressed in recent years. Its applications span industries such as digital entertainment, advertising, media, e-commerce, education, and healthcare [22]. In product design, AIGC enhances design efficiency and fosters innovation in both design solutions and processes [23].

The emergence of ChatGPT in 2022 marks the beginning of general-purpose AI [24]. As a prominent example of graph-generative AI, ChatGPT has become the fastest-growing application in history, powered by large language models (LLM). Commonly used image generation models include MidJourney, Stable Diffusion, and DALL-E. MidJourney utilizes a denoising algorithm based on bilateral filtering, requiring minimal model tuning and offering high-quality image generation [25]. Stable Diffusion, on the other hand, uses an image smoothing algorithm, with higher operational requirements and open-source access for various plugins [26]. DALL-E, integrated into OpenAI’s GPTs, leverages ChatGPT’s LLMs for highly accurate understanding of prompt words [27]. While video generation tools like SORA, Runway ML, and Dreamina exist, they are not explored here due to their limited relevance to product design.

Currently, AIGC in design primarily enhances efficiency by automating repetitive tasks and generating numerous solutions quickly and cost-effectively [28]. In product design, AIGC tools mainly serve as sources of modeling inspiration. While AIGC boosts productivity, it still requires designers to engage creatively and perform other tasks to achieve high-quality solutions [29]. Increasingly, researchers are exploring human-AIGC collaboration to optimize design efficiency and solutions through synergy [30]. Wang et al. suggest that designers can use AIGC-generated elements and templates to rapidly respond to customer needs, guide design through data analysis and trend prediction, and innovate collaboratively to inspire creativity and conceptual development [31]. Liao et al. further demonstrate that AIGC can overcome design barriers, stimulate creativity, and provide real-time suggestions and analyses [32].

In complex product conceptual design, the use of AIGC tools remains in its early stages. Most research is theoretical, with limited practical exploration. Research on AIGC applications in the product design process, especially in sustainable design, is still lacking. Additionally, AIGC-generated designs often lack originality, human aesthetic judgment, and are subject to data bias, model instability, and ethical issues. These challenges require ongoing research, with human oversight to manage AIGC input and output. This study aims to explore innovative solutions for outdoor cookware design. Building upon this exploration, it develops a specific AIGC-assisted product design approach. The product conceptual design process proposed in this study shares certain similarities with the methods of Pugh and Pahl & Beitz, in that it follows a structured approach starting from user needs, proceeding through concept generation and evaluation, and supporting systematic comparison of multiple design alternatives. However, unlike traditional methods, this approach introduces AIGC as an active collaborative participant to enable rapid idea generation. This significantly improves design efficiency and makes the design process more flexible, particularly for complex design problems.

Furthermore, by integrating AIGC with sustainable design considerations, the study proposes an innovative design process to support and advance sustainable product design practices.

## 3. Research framework

**Fig 1:**
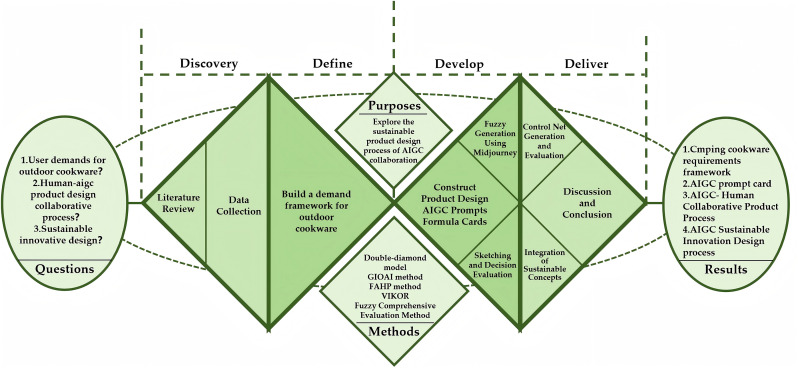
Design research framework based on the double diamond model.

This study adopts the Double Diamond model, a systematic methodology that enhances the rigor of the research process by organizing it into two iterative cycles of divergence and convergence. As the framework forms two consecutive diamond-shaped phases of divergence and convergence, it is therefore referred to as the Double Diamond model. The purpose of each phase is to identify and define the design problem, generate and validate the design solution, and ultimately ensure that the solution meets the user’s needs and goals [33]. As shown in Fig 1.

The “discovery” stage of the double-diamond model involves comprehensive research on camping cookware through literature review, field research, and internet searches. Information is systematically collected from online shopping platforms to identify camping cookware meeting specific requirements. Focus groups then screen and select representative camping cookware samples to serve as visual stimuli for subsequent interviews.

In the “define” stage, the focus shifts to constructing a user demand weight framework for camping cookware. Semi-structured interviews generate user requirement texts, which are then refined through the GIOIA method and clustered into Maslow’s five hierarchical levels to form dimensions. The Delphi method validates the dimensional model’s reasonableness, while FAHP assigns weights to all model elements. This framework provides scientific data support and theoretical foundations for subsequent innovative design and AIGC information input.

The “develop” stage leverages AIGC for creative exploration. AIGC prompts cards are constructed based on the camping cookware needs assessment framework, enabling precise reproduction and expression of product requirements and characteristics. To circumvent design fixation effects on idea generation, Midjourney generates fuzzy conceptual solutions. These numerous fuzzy solutions inform sketching processes, with VIKOR filtering the most reasonable solutions.

In the “delivery” stage, Stable Diffusion enables controlled expression and fuzzy comprehensive evaluation of design solutions. Sustainable design principles are integrated to develop innovative design pathways for camping cookware products. The research concludes with comprehensive review and discussion, examining the entire research process, proposing optimization suggestions, acknowledging limitations and shortcomings, and identifying future research directions.

## 4. Specific experimental process

### 4.1. Camping cookware user requirement analysis

#### 4.1.1. Sample collection of camping cookware.

**Fig 2:**
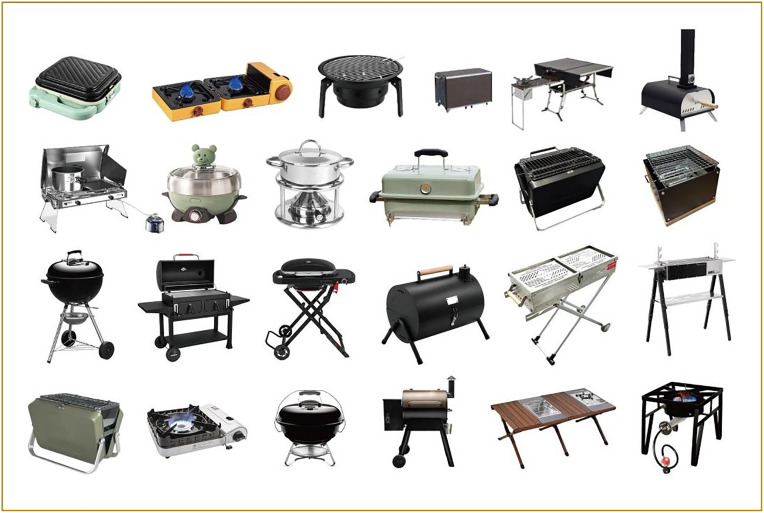
Camping cookware sample library.

In user interviews, image samples facilitate more specific expression of preferences and design pain points, enabling designers to identify latent needs and stimulate creative inspiration. Image samples were systematically collected from online shopping websites according to comprehensive rankings, with collection ceasing when samples began converging with previously collected items in appearance and functionality. To eliminate subjective influence from branding and background elements, all brand logos were concealed and backgrounds removed from images. This process yielded 23 images in Fig 2, establishing a comprehensive reference library of initial camping cookware samples.

**Fig 3:**
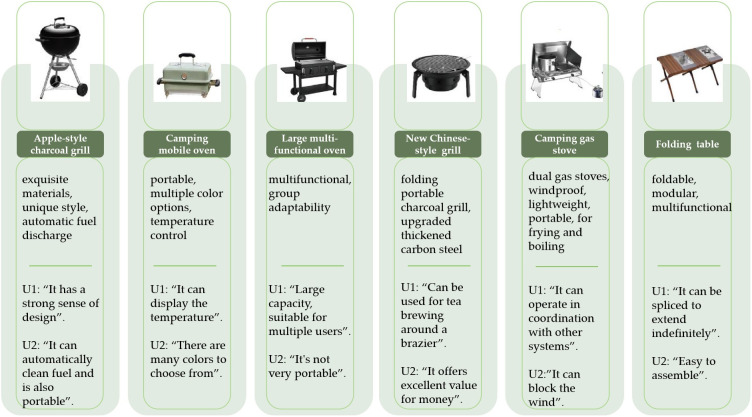
Formal experimental sample library.

An evaluation team consisting of product designers, master’s students in product design, and product design faculty members was established to further refine the images in the reference library of preliminary samples, compare the appearance criteria such as style, color, and material of the products. The selection of representative images follows the principles of representativeness and diversity to ensure coverage of the main types of design features. Six typical picture samples were finally selected, and the sample content mainly consisted of product pictures and descriptive introductions, as shown in Fig 3.

#### 4.1.2. Searching for user requirements.

Semi-structured interviews were conducted to gather users’ perspectives on ideal camping cookware, using the six previously mentioned samples. Participants were recruited via social media and student referrals, utilizing random and snowball sampling methods, resulting in 22 volunteers with a strong interest in outdoor activities. The number of participants was determined with reference to sample sizes commonly used in similar experimental studies, while balancing research resources and practical feasibility. Selection criteria included: (1) at least three previous camping experiences; (2) high standards for camping quality, particularly in cooking; and (3) diverse demographics, with a balanced gender ratio and varied occupations, income levels, and purchasing motivations. The interview outline, developed from literature review and focus group discussions, focused on user needs and sustainability concepts (as shown in S1 File).

Before the interviews, researchers explained the study’s purpose and obtained informed consent from all participants. Recognizing the importance of the interview atmosphere, quiet indoor venues were chosen for in-person sessions, while online meetings were held for remote participants. Researchers fostered a relaxed environment, followed the interview outline, asked relevant follow-up questions, and encouraged participants to provide detailed descriptions of their feelings and suggestions. Each 15-minute interview was recorded, and after the session, the audio was transcribed and cross-referenced with interview notes to compile comprehensive texts reflecting users’ visions for ideal camping cookware.

#### 4.1.3. Analyzing user requirements.

Based on user interview data, this study applies the GIOIA method to systematically analyze and cluster findings according to Maslow’s five levels of needs. Developed by Dennis A. Gioia and colleagues, this approach provides a structured and transparent framework for qualitative analysis, enabling a clear progression from raw data to theoretical constructs. Compared with other methods such as grounded theory, thematic analysis, and content analysis, the GIOIA method offers greater systematicity in data structuring and concept development, making it particularly suitable for exploratory research. Therefore, it is adopted in this study to enhance analytical rigor and traceability, and its effectiveness has been widely validated in prior research. The methodology consists of three stages: (1) Primary coding, which categorizes data to extract users’ key requirements and visions; (2) Secondary coding, which generalizes key needs from primary coding to identify higher-level need keywords; and (3) Need element clustering, which organizes these keywords into dimensions. This study clusters elements based on Maslow’s hierarchy, resulting in a dimensional system encompassing functional, safety, collaborative, emotional, and experiential needs. Tables 1 and 2 illustrate the GIOIA clustering process for specific user interview content.

**Table 1:** Clustering process for specific user interview content 1.

Original Interview Statements (First-Order Concepts)	Second-Order Themes	Aggregation Dimension
“I hope outdoor cookware can be multi-functional and integrated. It shouldn’t be limited to grilling meat or cooking.	Multiple cooking functions	Functional requirements
“I like this one. It feels easy to operate. Although many products are very convenient, they are very troublesome to assemble. I would rather it be a bit heavier than spend time figuring out how to assemble it.”	Usability	
“I hope outdoor cookware can be portable and must have a handle. The green one in the picture would be great.”	Portability	
“It is hoped to conform to the principles of ergonomics.”	Ergonomic adaptability	
“I hope the height is adjustable.”		
“I hope the cooker can automatically control temperature or operate autonomously, allowing me to enjoy nature instead of spending time cooking.”	Intelligent Cooking Assistance	
“Material safety is very important. Do not release toxic gases after heating.”	Material safety	Safety requirements
“Safety is of the utmost importance to me. I get scared whenever I see an external gas stove, especially in an outdoor environment.”	Safety protection system	
“If it’s charcoal grilling, I hope there could be a windscreen. It’s very difficult to light the charcoal when the wind is strong when cooking outdoors.”		
“Cooking utensils should be equipped with slots for first aid kits. Outdoor accidents can occur at any time.”	Emergency monitoring system	
“I hope the product is relatively durable and has a relatively stable structure.”	Structural stability	
“I hope my product has certain social functions, such as being able to see who is also using the products of the same brand within a certain range and establishing social connections.”	Social attribute	Collaborative requirements
“I hope to develop a dedicated community to find like-minded camping enthusiasts who can share their cooking achievements, campsite locations or event invitations.”		
“I hope it can be used by partners together. It can be improved in terms of function and structure to adapt to the simultaneous use of two or more people.”	Collaborative usage	
“The family and friends can use it together, hoping that the product can meet the dining needs of many people.”	Group adaptability	

**Table 2:** Clustering process for specific user interview content 2.

Original Interview Statements (First-Order Concepts)	Second-Order Themes	Aggregation Dimension
“Using eco-friendly materials is a responsibility we all share.”	Sustainable consideration	Emotional requirements
“Eco-friendly fuel matters to me as an environmentalist.”		
“Can solar panels be integrated on the surface of cookware? Outdoor charging is too important.”		
“I hope to have a higher level of appearance. It would be best if it could reflect my personality and aesthetic sense. Appearance is very important. I really don’t like all the black and white colors in the picture. I wouldn’t even give it a second glance.”	Aesthetic appeal	
“I think if the product can highlight my own individual needs, I will choose to purchase it.”	Personalized customization	
“Brand collaborations or co-branding could enhance uniqueness.”	Brand cross-border integration	
“Be able to accommodate the dietary habits of different groups of people.”	Cultural resonance	
“I hope to be able to meet different scenarios, such as outdoor riverside, mountainside, park and other different places.”	Multi-scene compatibility	Experiential requirements
“Smart features like a touchscreen would improve interaction.”	Intelligent interaction	
“I hope to achieve interaction and feedback through means such as sound, vision, or touch.”	Multi-sensory interaction	
“I hope to be able to remotely control the heat through the mobile phone APP and even preheat the cooking utensils in advance.”	Entertainment experience system	
“I have high-quality requirements for camping. I hope it have music. “		

#### 4.1.4. Constructing an indicator framework.

After the user requirements clustering is finished, the clustering results need to be evaluated for reasonableness. Therefore, we invited 5 university professors and 5 product designers to set up an expert evaluation team. 5 university professors have rich experience in product teaching and practice, and 5 product designers have been practicing for more than 5 years, participated in a number of product design and development, and have won many international design awards. Expert information is shown in Table 3. Two rounds of surveys were conducted using the Delphi method to obtain professional insights from expert perspectives to refine the assessment framework.

**Table 3:** Expert information form.

Item	Indicator	Frequency	Percentage (%)
Gender	Male	5	50
	Female	5	50
Age	20-29	3	30
	30-39	3	30
	40-49	2	20
	50-59	2	20
Education Level	Bachelor’s degree	3	30
	Master’s degree	2	20
	Doctor’s degree	5	50
Occupation	University Professor	5	50
	Designer	5	50

The experiment was conducted through online channels. Experts were invited to evaluate the above proposed clustering of user requirements and express their opinions, and the overall experimental process was carried out through anonymity. After the first round, based on the statistical analysis and expert feedback, the findings were integrated and improved, and feedback was provided anonymously. In the second round, the above is continued and repeated, and the process is concluded after the experts reach a consensus. The Delphi method utilizes expert knowledge and experience by allowing each participant to independently assess the problem and develop a final consensus through repeated feedback.

**Fig 4:**
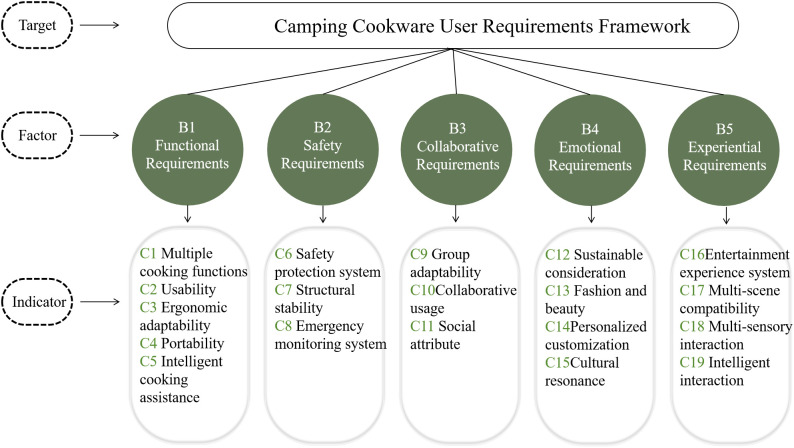
Camping cookware user requirements framework.

After expert consensus was reached, a framework of user requirements for camping cookware was developed based on Maslow’s hierarchy of needs. This framework categorizes user inputs into different levels, enabling a structured understanding of needs from basic functional requirements to higher-level emotional and value-related aspects. Such classification helps clarify design priorities at the conceptual stage and provides more structured inputs for AIGC, thereby improving the relevance and rationality of generated design concepts. Considering that this study focuses on the conceptual design stage, factors such as material properties, branding, and marketing strategies are difficult to quantify and are not easily translated into creative design elements, and were therefore excluded. Finally, a design system consisting of 19 evaluation indicators across six dimensions was established, as shown in Fig 4.

#### 4.1.5. Assignment of user requirements weights.

We employ FAHP for user requirements weight assignment, which integrates fuzzy theory with hierarchical analysis. Fuzzy theory addresses imprecise data and solves decision-making problems with fuzzy attributes [34]. Unlike traditional hierarchical analysis, FAHP accounts for uncertainty in human perception and more accurately represents preferences through fuzzy numbers [35]. FAHP has gained widespread application across design fields, including product design [36], fashion design [37], and arts and crafts [38].

An expert panel participates in a research questionnaire constructed using fuzzy triangular functions, conducting pairwise comparisons and scoring the relative importance of each element. Scoring results serve as the median values of triangular functions, with judging criteria referenced and the upper and lower bounds of fuzzy triangular functions follow values (as shown in S2 File). After scoring, experts assess their confidence levels.The specific calculation process is shown below.

Step 1: Construct the judgment matrix

Construct a triangular fuzzy judgment matrix A,where $a\begin{array}{c} ij \end{array}=\left(l_{ij},m_{ij},u_{ij}\right)$, $a\begin{array}{c} ij \end{array}$ is a closed interval with $m_{ij}$ as its median:


$$\begin{array}{c} A=(a_{ij}{)}_{n\times n} \end{array}$$ (1)

Calculate the geometric mean of the numbers at each position of all triangular fuzzy judgment matrices to obtain the final triangular fuzzy judgment matrix. Among them, $\left(l_{ij}^{k},m_{ij}^{k},u_{ij}^{k}\right)$ is the triangular fuzzy number representing the importance score given by the k expert for indicator i relative to indicator j, and n is the number of experts:


$$\begin{array}{c} \left({\overset{―}{l}}_{ij},\text{\textbackslash{}hspace\{0.33em}\}{\overset{―}{m}}_{ij},\text{\textbackslash{}hspace\{0.33em}\}{\overset{―}{u}}_{ij}\right)=\left(\sqrt[{n}]{l_{ij}^{\text{1}}l_{ij}^{\text{2}}\cdots l_{ij}^{n}},\text{\textbackslash{}hspace\{0.33em}\}\sqrt[{n}]{m_{ij}^{\text{1}}m_{ij}^{\text{2}}\cdots m_{ij}^{n}},\text{\textbackslash{}hspace\{0.33em}\}\sqrt[{n}]{u_{ij}^{\text{1}}u_{ij}^{\text{2}}\cdots u_{ij}^{n}}\right) \end{array}$$ (2)

Construct a fuzzy evaluation factor matrix E, where $e_{ij}=\frac{u_{ij}-l_{ij}}{2m_{ij}}$ is the standard deviation rate:


$$\begin{array}{c} E=(e_{ij}{)}_{n\times n}=\left(\begin{array}{cccc} @cccc@1 & 1-\frac{u_{12}-l_{12}}{2m_{12}} & \cdots & 1-\frac{u_{1n}-l_{1n}}{2m_{1n}}\\ 1-\frac{u_{21}-l_{21}}{2m_{21}} & 1 & \cdots & 1-\frac{u_{2n}-l_{2n}}{2m_{2n}}\\ \vdots & \vdots & \ddots & \vdots \\ 1-\frac{u_{n1}-l_{n1}}{2m_{n1}} & 1-\frac{u_{n2}-l_{n2}}{2m_{n2}} & \cdots & 1 \end{array}\right) \end{array}$$ (3)

Calculate the adjustment judgment matrix Q, convert the calculated Q into columns $Q^{\prime }$:


$$\begin{array}{c} Q=M\times E=\left[\begin{array}{cccc} @cccc@m_{11} & m_{12} & \cdots & m_{1n}\\ m_{21} & m_{22} & \cdots & m_{2n}\\ \vdots & \vdots & \ddots & \vdots \\ m_{n1} & m_{n2} & \cdots & m_{nn} \end{array}\right]\times \left[\begin{array}{cccc} @cccc@1 & 1-\frac{u_{12}-l_{12}}{2m_{12}} & \cdots & 1-\frac{u_{1n}-l_{1n}}{2m_{1n}}\\ 1-\frac{u_{21}-l_{21}}{2m_{21}} & 1 & \cdots & 1-\frac{u_{2n}-l_{2n}}{2m_{2n}}\\ \vdots & \vdots & \ddots & \vdots \\ 1-\frac{u_{n1}-l_{n1}}{2m_{n1}} & 1-\frac{u_{n2}-l_{n2}}{2m_{n2}} & \cdots & 1 \end{array}\right] \end{array}$$ (4)

Step 2: Calculate the weights of each indicator using the square root method.

Calculate the n root of all elements in each row:


$$\begin{array}{c} {\overset{―}{\omega }}_{i}={\left(\prod_{j=1}^{n}a_{ij}\right)}^{\frac{\text{1}}{n}},\text{\textbackslash{}hspace\{1em}\}i=1,2,\cdots ,n \end{array}$$ (5)

Normalize ${\overset{―}{\omega }}_{i}$:


$$\begin{array}{c} \omega_{i}=\frac{{\overset{―}{\omega }}_{i}}{\sum_{i=1}^{n}{\overset{―}{\omega }}_{i}},\text{\textbackslash{}hspace\{1em}\}i=1,2,\cdots ,n \end{array}$$ (6)

Calculate the final weight:


$$\begin{array}{c} W=\left[\omega_{\text{1}},\omega_{\text{2}},\cdots ,\omega_{n}\right] \end{array}$$ (7)

Step 3: Consistency testing.

Calculate the maximum eigenvalue of the median matrix $\lambda_{max}$:


$$\begin{array}{c} \lambda_{max}=\sum_{i=1}^{n}\frac{(PW{)}_{i}}{nW_{i}}\text{\textbackslash{}hspace\{1em}\}(i,j=1,2,3,\ldots ,n). \end{array}$$ (8)

Determine consistency indicators:


$$\begin{array}{c} CI=\frac{\lambda_{max}-n}{n-1} \end{array}$$ (9)

According to the RI table for the corresponding order, we obtain RI, If the calculation result is CR<0.1, then the consistency test is passed:


$$\begin{array}{c} CR=\frac{CI}{RI} \end{array}$$ (10)

Since this study employs a multi-expert collaborative scoring method, it is necessary to calculate the geometric mean of each position in the triangular fuzzy matrix formed after all experts have scored. The final geometric mean triangular fuzzy judgment matrix for the five requirements is obtained using Formula (1)-(2), as shown in Tables 4–9.

**Table 4:** Geometric mean matrix of factor layer requirements.

B1	B2	B3	B4	B5
(1.000, 1.000, 1.000)	(0.903, 1.246, 1.635)	(3.697, 4.258, 4.802)	(3.516, 4.072, 4.613)	(0.815, 1.046, 1.437)
(0.611, 0.803, 1.108)	(1.000, 1.000, 1.000)	(1.660, 2.169, 2.724)	(2.237, 3.178, 4.095)	(0.617, 0.802, 1.118)
(0.208, 0.235, 0.270)	(0.367, 0.461, 0.602)	(1.000, 1.000, 1.000)	(1.021, 1.552, 2.048)	(0.407, 0.485, 0.595)
(0.217, 0.246, 0.285)	(0.244, 0.314, 0.446)	(0.488, 0.644, 0.979)	(1.000, 1.000, 1.000)	(0.383, 0.451, 0.544)
(0.696, 0.956, 1.227)	(0.894, 1.245, 1.621)	(1.682, 2.062, 2.460)	(1.837, 2.215, 2.612)	(1.000, 1.000, 1.000)

**Table 5:** Functional requirements geometric mean matrix.

C1	C2	C3	C4	C5
(1.000, 1.000, 1.000)	(0.470, 0.608, 0.813)	(0.360, 0.474, 0.607)	(0.669, 0.802, 0.952)	(2.495, 3.004, 3.519)
(1.230, 1.643, 2.128)	(1.000, 1.000, 1.000)	(0.907, 1.262, 1.598)	(0.896, 1.134, 1.455)	(4.306, 4.829, 5.346)
(1.649, 2.112, 2.773)	(0.626, 0.792, 1.103)	(1.000, 1.000, 1.000)	(1.370, 1.844, 2.296)	(4.345, 5.078, 5.695)
(1.051, 1.245, 1.495)	(0.687, 0.882, 1.116)	(0.436, 0.542, 0.730)	(1.000, 1.000, 1.000)	(2.832, 3.758, 4.571)
(0.284, 0.333, 0.401)	(0.187, 0.207, 0.232)	(0.176, 0.197, 0.230)	(0.219, 0.266, 0.353)	(1.000, 1.000, 1.000)

**Table 6:** Safety requirements geometric mean matrix.

C6	C7	C8
(1.000, 1.000, 1.000)	(0.836, 1.262, 1.657)	(0.620, 0.870, 1.164)
(0.603, 0.792, 1.196)	(1.000, 1.000, 1.000)	(1.076, 1.496, 1.966)
(0.859, 1.148, 1.613)	(0.509, 0.668, 0.929)	(1.000, 1.000, 1.000)

**Table 7:** Collaborative requirements geometric mean matrix.

C9	C10	C11
(1.000, 1.000, 1.000)	(0.469, 0.545, 0.625)	(1.513, 1.864, 2.295)
(1.597, 1.835, 2.135)	(1.000, 1.000, 1.000)	(3.237, 3.764, 4.286)
(0.436, 0.537, 0.661)	(0.233, 0.266, 0.309)	(1.000, 1.000, 1.000)

**Table 8:** Emotional requirements geometric mean matrix.

C12	C13	C14	C15
(1.000, 1.000, 1.000)	(2.552, 2.930, 3.337)	(2.418, 2.786, 3.185)	(1.938, 2.352, 2.865)
(0.300, 0.342, 0.392)	(1.000, 1.000, 1.000)	(1.879, 2.551, 3.173)	(1.879, 2.551, 3.173)
(0.314, 0.359, 0.414)	(0.315, 0.392, 0.532)	(1.000, 1.000, 1.000)	(1.349, 1.782, 2.199)
(0.348, 0.425, 0.516)	(0.315, 0.392, 0.532)	(0.454, 0.561, 0.741)	(1.000, 1.000, 1.000)

**Table 9:** Experiential requirements geometric mean matrix.

C16	C17	C18	C19
(1.000, 1.000, 1.000)	(0.622, 0.724, 0.847)	(0.379, 0.450, 0.540)	(0.250, 0.299, 0.377)
(1.180, 1.379, 1.609)	(1.000, 1.000, 1.000)	(0.863, 1.246, 1.679)	(0.433, 0.549, 0.708)
(1.853, 2.221, 2.642)	(0.596, 0.803, 1.158)	(1.000, 1.000, 1.000)	(0.273, 0.370, 0.603)
(2.654, 3.347, 4.006)	(1.413, 1.820, 2.308)	(1.657, 2.702, 3.665)	(1.000, 1.000, 1.000)

Subsequently, the relative weights of each factor are calculated using Formula (3)-(7). Additionally, consistency testing is performed on the evaluation matrix using Formula (8)-(10). The CR value for the five factor layers is 0.016 < 0.1, indicating that the consistency test is passed. Furthermore, the CR values for all sub-indicator evaluation matrices are also less than 0.1. When aggregated, as shown in the table, this demonstrates that the construction and results of the evaluation matrices are reasonable. As shown in Table 10.

**Table 10:** Weighting and consistency checks.

Factor	Weight	Indicator	Weight	Consistency check
B1	0.348	C1	0.164	CR = 0.009 < 0.1
		C2	0.274	
		C3	0.3	
		C4	0.206	
		C5	0.056	
B2	0.238	C6	0.339	CR = 0.064 < 0.1
		C7	0.356	
		C8	0.305	
B3	0.111	C9	0.289	CR = 0.001 < 0.1
		C10	0.559	
		C11	0.153	
B4	0.079	C12	0.425	CR = 0.076 < 0.1
		C13	0.3	
		C14	0.164	
		C15	0.111	
B5	0.224	C16	0.125	CR = 0.022 < 0.1
		C17	0.208	
		C18	0.223	
		C19	0.444	

#### 4.1.6. Requirements weighting analysis.

**Fig 5:**
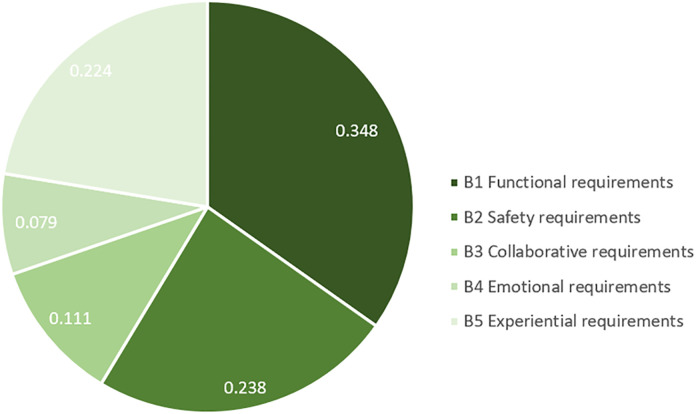
Factor layer requirements weighting.

Fuzzy hierarchical analysis results reveal that among the five Maslow needs for outdoor cookware, functional requirements carry the highest weight (0.348), establishing them as the primary consideration and reflecting cookware’s essential attributes as survival tools in outdoor scenarios. Safety requirements rank second (0.238), demonstrating users’ emphasis on cookware safety, particularly in complex natural environments with rigid requirements for scald prevention, fire prevention, and stability. Experiential requirements (0.224) follow closely, indicating product upgrade directions and differentiation opportunities through intelligent features and multi-sensory interactions that enhance user experience. Although collaborative requirements (0.111) and emotional requirements (0.079) receive lower weights, they remain significant—the former addressing camping economy’s social attribute extensions, while the latter relates to products’ visual aesthetics and emotional resonance. The specific weight distribution results are illustrated in Fig 5.

In terms of functional requirements, ergonomics adaptability (0.3) is given the highest priority. The design must strictly prioritize the user experience, optimizing it to align with human physiological, psychological characteristics, and behavioral habits, thereby enhancing efficiency, comfort, and safety. This should be done in conjunction with the user’s outdoor usage scenarios and operational habits, while also balancing the relationship between functionality and design. Appropriate design methods such as modularization and minimalism should be selected. The weighting rankings for other elements are as follows: usability (0.274)> portability (0.206)> multiple cooking functions (0.164)> intelligent cooking assistance (0.056). Regarding product safety requirements, structural stability (0.356) is the most important factor, indicating that it is the fundamental prerequisite for ensuring user safety. Followed by product safety protection system (0.339)> emergency monitoring system (0.305), ensuring safety during use. In collaborative requirements, collaborative usage (0.559) is the most critical, stemming from young people’s pursuit of cooperation and sharing in outdoor social settings, enhancing interactive fun and fostering emotional connections through collaborative cooking processes. The remaining two elements are ranked as group adaptability (0.289)> social attribute (0.153). In terms of emotional requirements, sustainability has the highest weighting (0.425). As the social environment deteriorates, users are more inclined to choose sustainable alternatives, achieving self-worth through supporting sustainable behaviors and responding to society’s collective calls for ecological protection. This is followed by aesthetic appeal (0.3). In the context of charming camping, camping cookware has become an aesthetic carrier that combines functionality and emotional value, greatly enhancing users’ psychological satisfaction and desire for social display. The remaining two elements are ranked as follows: personalized customization (0.164)> cultural resonance (0.111). In terms of experiential requirements, intelligent interaction has the highest weighting (0.444), driven by users’ demand for upgraded social and personalized experiences enabled by technology in the charm camping outdoor setting. Multi-sensory interaction (0.223)> multi-scene compatibility (0.208)> entertainment system (0.125).

**Fig 6:**
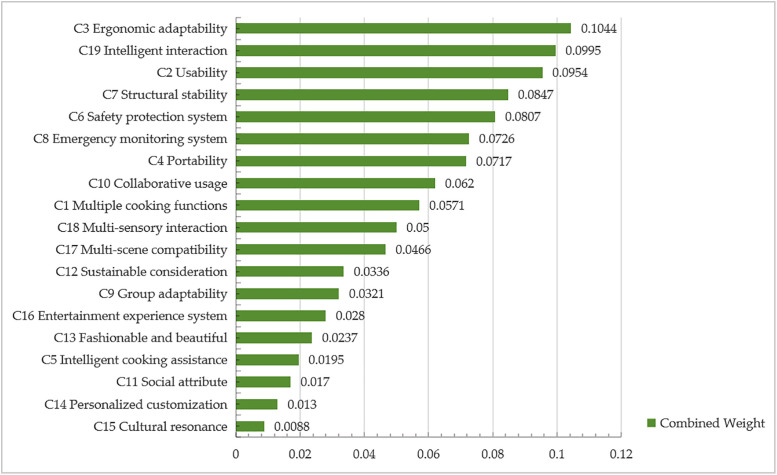
Comprehensive weighted ranking results.

Integrating factor and indicator layer weights yields comprehensive rankings as shown in the figure. Ergonomic adaptability (C3 0.1044) ranks first, with other functional requirements also ranking highly: usability (C2, 0.0954) and portability (C4, 0.0717). This demonstrates that simplicity and ease of operation are key priorities for camping cookware, particularly in outdoor scenarios where users demand higher convenience levels, preferring to spend more time enjoying outdoor activities rather than cooking. Intelligent interaction (C19, 0.0995), ranking second, reflects both the need for smart interactive technology and indirectly emphasizes convenience requirements. Within safety attributes, structural stability (C7,0.0847), safety protection system (C6,0.0807), and emergency monitoring system (C8,0.0726) rank prominently, confirming safety as a critical concern for cooking equipment. Additionally, social collaboration and multifunctional assistance emerge as key priorities, particularly in social scenarios where cookware assumes social attributes, enhancing emotional connections through collaborative interaction. The specific weight distribution results are shown in Fig 6.

In contrast, aesthetic appeal, social attribute, personalized customization, and emotional requirements receive lower weights, making them less likely to serve as key user need priorities. However, these comprehensive assessment results are influenced by indicator layer evaluations, and certain factor layer elements may yield different results when separated from indicator layer influence. Therefore, while comprehensive assessment serves as a reference factor, it cannot function as the sole reference, requiring case-specific analysis. These results provide clear guidance for prioritizing camping cookware design by emphasizing usability, smart interactive features, safety, and collaborative operation concerns while appropriately simplifying lower-priority features. This study offers theoretical reference for designers developing camping cookware designs.

### 4.2. Generative AI-assisted design process

#### 4.2.1. Creating prompts formula cards.

Prompts are textual content inputted by the user during interaction with generative AI to convey instructions and requests to the model so that the model recognizes and responds to them [39]. Numerous studies have shown that the quality of the images generated by the model is greatly influenced by the quality of the prompts. Through the literature review and the Midjourney comparison test, we found that the arrangement and presentation order of prompts are crucial, and in general, the more advanced the cue words are, the greater the impact on the image generation results [40]. At the same time, the prompts should be accurate and comprehensive, through the standardization of the prompts formula cards to regulate the process of image generation, to reduce the cost of trial and error in the process of design practice, to help designers to quickly and efficiently improve the quality of generative AI-generated images. The prompts formula cards is shown as follows: reference image + target product + main details (shape, color, decoration, material) + viewpoint +stylism + background + light + clarity + parameters.

**Fig 7:**
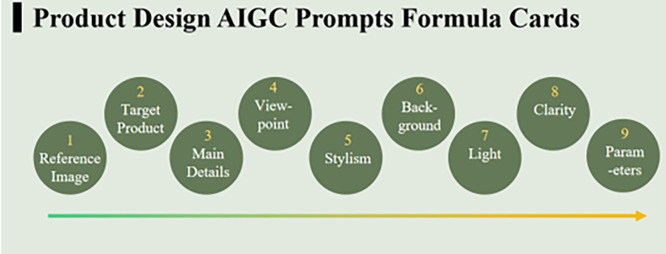
Product design AIGC prompts formula cards.

Using Midjourney as an experimental tool, the prompts formula cards is proposed to assist product generation, which is not only applicable to the camping cookware studied in this paper, but also to other product development. In addition, generative AIs such as stable diffusion and DALL-E3 are also applicable. In order to make the formula to understand and visualize, we constructed an application card as shown in Fig 7.

In order to verify the validity of the prompt cards, we conducted five sets of control experiments and invited five participants, all of whom were second-year master’s students majoring in product design with experience in using AIGC and assisting in product design. Each group had one participant who needed to complete two AIGC generation tasks. To prevent the prompt card formulas from influencing the participants’ free-form thinking, all participants were asked to first perform free-form tasks, followed by strictly adhering to the prompt word process for image generation. The experiment was conducted under the supervision of staff. After the experiment, an expert panel was invited to evaluate the results using a seven-point Likert scale, assessing based on reference image fidelity, generated image quality (visual coherence, color harmony, product structural rationality, and stylistic diversity), and semantic relevance. Among these, image restoration degree refers to the extent to which the generated image preserves the features of the original input; image quality focuses on clarity, completeness, and overall visual performance; and semantic relevance measures the consistency between the generated results and the user’s requirements or input semantics. The comprehensive evaluation results are shown in Table 11 below.

**Table 11:** AIGC comparison generation scorecard.

Participant	Frequency	Image restoration degree	image quality	Semantic relevance
Designer A	1	4.9	4.7	4.5
	2	5.9	5.8	5.8
Designer B	1	4.8	5.0	4.6
	2	5.9	5.5	6.1
Designer C	1	4.6	4.6	4.7
	2	5.9	5.4	5.4
Designer D	1	5.8	5.8	5.6
	2	5.7	5.7	6.0
Designer E	1	4.9	4.9	4.7
	2	5.7	5.6	6.1

Overall, images generated using prompt words outperformed freely generated images across all three metrics. User D had some unexpected results, with the indicators of the two experiments being very similar, image restoration degree and image quality generated was better than that generated using prompt cards. We immediately interviewed this participant and learned that he was very interested in AIGC and had his own prompt input logic. He also agreed with the prompt cards proposed by this study, believing that important prompts should be placed at the beginning, as this would affect the output results. Given the randomness of AIGC, there is no absolute good or bad when it comes to prompt cards, and multiple experiments are needed to find the appropriate input method.

Therefore, we concluded that the prompts cards have a certain value and can reasonably control the input of prompt words, improving the quality and fidelity of the generated images through formatting, which is very useful for AIGC beginners, but not the best for those who are skilled in the field of AIGC. Therefore, the composition of prompt words can be further improved through more experiments. A more objective method can also be used to evaluate the quality of the generated images, increasing their persuasiveness. The subsequent outdoor camping cookware design practice in this study was conducted in strict accordance with the guidance of the above prompt formula cards.

#### 4.2.2. Midjourney fuzzy generation.

Thanks to the optimization of the diffusion model algorithm and the establishment of large-scale and training sets, generative artificial intelligence can generate a large number of high-quality image materials in a short period of time [41]. This provides designers with a steady stream of design solutions for reference in the preliminary demand analysis and thought diffusion stages [42]. This design solution is in some ways extremely consistent with the final design output, and is therefore also known as a high-fidelity pre-solution. Premature exposure to high-fidelity solutions in the pre-design phase will, to a certain extent, produce design fixation [43]. Design fixation refers to the tendency of designers to add designs or elements contained in pictures to their own designs when dispersing creative ideas through picture design examples [44], the fundamental reason being that the visual stimulus of high-fidelity pictures contains too much information and is too specific [45].

**Fig 8:**
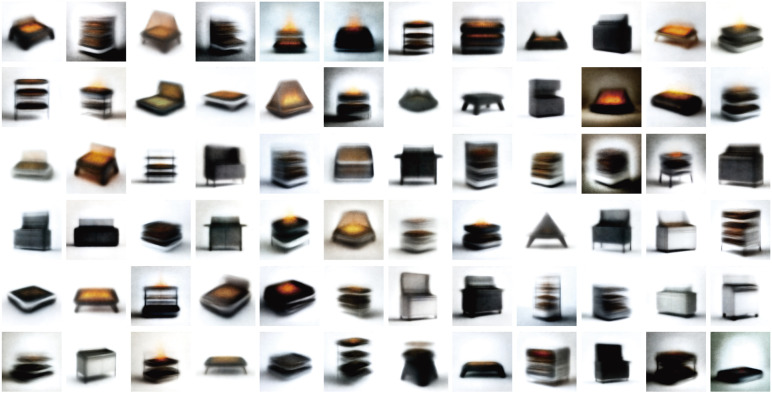
The Final Generated Fuzzy Map.

**Fig 9:**
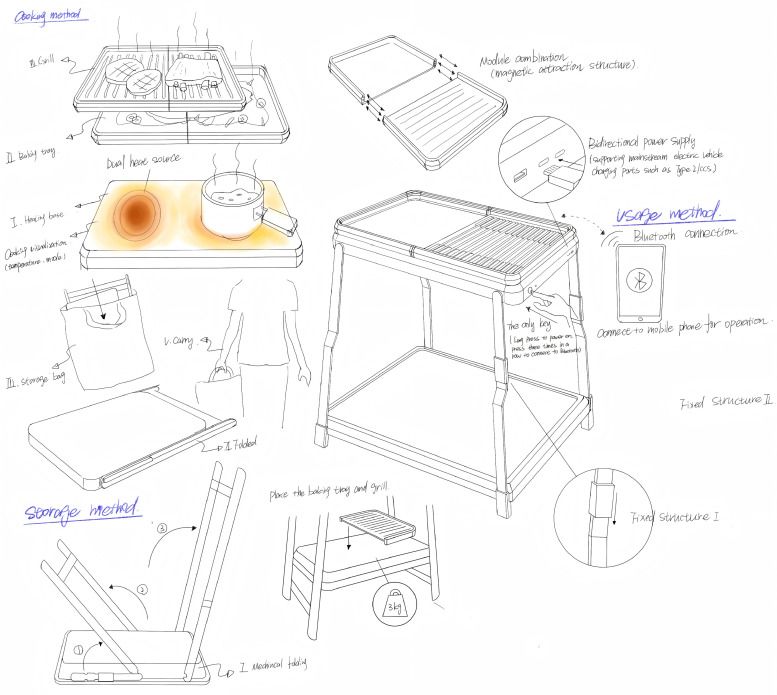
Schematic diagram of Plan A.

**Fig 10:**
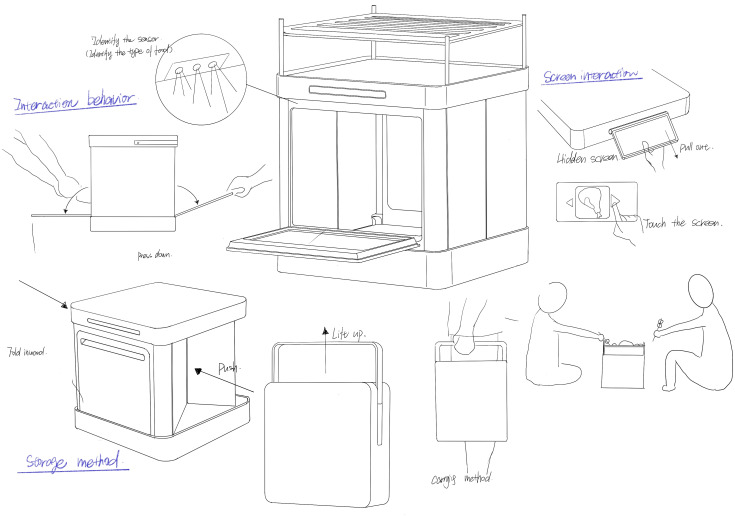
Schematic diagram of Plan B.

**Fig 11:**
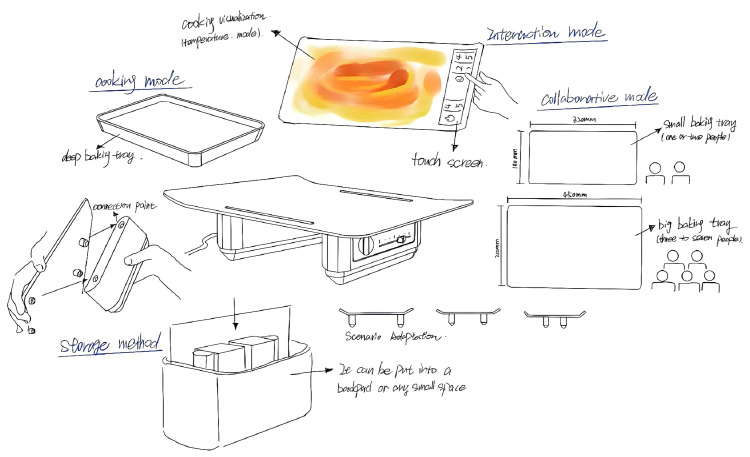
Schematic diagram of Plan C.

In order to avoid the design fixation caused by the overly concrete visual stimuli, Mid journey’s fuzzy generation is used to enhance the strengths and avoid the weaknesses by blurring the degree of concrete visual stimuli. The reason for choosing Midjourney as the blur generation is that Midjourney is better than Stable Diffusion in terms of control lability of blur production and accuracy of cue word understanding, and the blur image is generated by using the “-stop” parameter setting, and the “-stop” parameter setting is used to generate the blur image, and the “-stop” parameter setting is used to generate the blur image. The number “n” after the “-stop” parameter is the degree of completion of the generated image, which takes the value from 10 to 100, and finally “n” is chosen to be 10 for blurring. The prompts words are referred to the prompts formula cards proposed in the previous stage, and the weight assignment results of the camping cookware requirements framework are used to filter the demands and input them from the front to the back according to the size of the weight assignment. The final generated fuzzy map ensemble is shown in Fig 8. Based on the stimulus of the fuzzy graph, the sketch of camping cookware is drawn, and three scenarios are drawn, as shown in Figs 9, 10, and 11.

#### 4.2.3. VIKOR method for preferential decision making.

The evaluation of alternatives was conducted using the VIKOR method, a multi-attribute compromise ranking algorithm. This approach derives optimal solutions by combining the maximization of group utility and the minimization of individual regret to establish a compromise ranking among alternatives. Furthermore, VIKOR generates not only a unique optimal solution but also a compromise solution set under mutual concession among stakeholders. This capability makes VIKOR particularly suitable for multi-stakeholder, multi-objective product decision-making, enabling the identification of optimal comprehensive compromise solutions from multiple alternatives. The calculation flow is shown below

There are usually m alternatives in the product development phase, and an initial matrix F is obtained by scoring each alternative based on the n evaluation metrics established in the FAHP-Delphi method:


$$\begin{array}{c} F=\left[\begin{array}{cccc} @cccc@f_{11} & f_{12} & \cdots & f_{1n}\\ f_{21} & f_{22} & \cdots & f_{2n}\\ f_{m1} & f_{m2} & \cdots & f_{mn} \end{array}\right] \end{array}$$

The initial evaluation matrix was normalized by using (11) for cost-based indicators and (12) for benefit-based indicators, and where i denotes the i option and j denotes the j evaluation indicator corresponding to the option, $f_{ij}$ is the evaluation value of the i alternative for the j evaluation indicator.


$$\begin{array}{c} e_{ij}=\frac{f_{ij}-min\left(f_{ij}\right)}{max\left(f_{ij}\right)-min\left(f_{ij}\right)}\text{\textbackslash{}hspace\{1em}\} \end{array}$$ (11)

$$\begin{array}{c} e_{ij}=\frac{max\left(f_{ij}\right)-f_{ij}}{max\left(f_{ij}\right)-min\left(f_{ij}\right)}\text{\textbackslash{}hspace\{1em}\} \end{array}$$ (12)

Based on (13) and (14), calculate the group utility value of the alternative $S_{\text{i}}$ and individual regret value $R_{\text{i}}$,where ${max}_{i}\left(e_{ij}\right)$ and ${min}_{i}\left(e_{ij}\right)$ are the maximum and minimum values of each column in the normalized matrix.


$$\begin{array}{c} S_{i}=\sum_{j=1}^{n}w_{j}\frac{{max}_{i}\left(e_{ij}\right)-e_{ij}}{{max}_{i}\left(e_{ij}\right)-{min}_{i}\left(e_{ij}\right)} \end{array}$$ (13)

$$\begin{array}{c} R_{i}={max}_{j}\left\{\frac{w_{j}({max}_{i}(e_{ij})-e_{ij})}{{max}_{i}\left(e_{ij}\right)-{min}_{i}\left(e_{ij}\right)}\right\} \end{array}$$ (14)

Calculate the compromise value $Q_{\text{i}}$ according to (15). Where the u value represents the coefficient of the decision-making mechanism, which generally defaults to 0.5


$$\begin{array}{c} Q_{i}=\frac{u(S_{i}-{min}_{i}S_{i})}{{max}_{i}S_{i}-{min}_{i}S_{i}}+\frac{\left(1-u)(R_{i}-{min}_{i}R_{i}\right)}{{max}_{i}R_{i}-{min}_{i}R_{i}} \end{array}$$ (15)

First, the alternatives are ranked from smallest to largest based on the values of $S_{\text{i}}$, $R_{\text{i}}$ and $Q_{\text{i}}$, with the smallest value being the preferred alternative. The sorting result is obtained as $Y_{\text{1}}$, $Y_{\text{2}}$, $Y_{\text{3}}$,…, $Y_{m}$. The sorting results are to be judged on the basis of the following conditions. Condition 1: $Q\left(Y_{\text{1}}\right)-Q\left(Y_{\text{2}}\right)\geq 1/(m-1)$. Condition 2: $Y_{i}$ takes the smallest value in at least one of the $S_{i}$ or $Q_{i}$ values. If the above conditions are satisfied by all, then $Y_{\text{1}}$ is optimal. If only condition 1 is satisfied then both $Y_{\text{1}}$ and $Y_{\text{2}}$ are compromises. If only condition 2 is satisfied, the maximum value of m is determined by the $Q\left(Y_{\text{1}}\right)-Q\left(Y_{\text{2}}\right)\geq 1/(m-1)$, which ultimately determines the compromise solution set.

According to the framework of indicators for the demand for camping cookware, 10 members of the Expert Assessment Panel were invited to assign values to a total of 19 evaluation indicators for each of the three alternatives using a 7-level scale, and their averages were calculated to form an initial evaluation matrix, as shown in Table 12, with all evaluation indicators of the type of benefit.

**Table 12:** Initial evaluation matrix.

Evaluation Dimension	Corresponding Metric	Solution1	Solution2	Solution3	Metric Type
Functional Requirements	Multiple cooking functions	6.6	4.4	5	Benefit criterion
	Usability	5.8	4.2	5	Benefit criterion
	Ergonomic adaptability	6.2	4.7	4.5	Benefit criterion
	Portability	6.7	5.7	5.6	Benefit criterion
	Intelligent Cooking Assistance	6	5.1	5	Benefit criterion
Safety Requirements	Safety protection system	5.3	6	5.8	Benefit criterion
	Structural stability	6	5.1	4.3	Benefit criterion
	Emergency monitoring system	5	4.1	3.8	Benefit criterion
Collaborative Requirements	Group adaptability	6	5	4.1	Benefit criterion
	Collaborative usage	6	5	3.8	Benefit criterion
	Social attribute	6.9	5	5	Benefit criterion
Emotional Requirements	Sustainable consideration	6	6	3.9	Benefit criterion
	Aesthetic appeal	6.4	4.3	4.9	Benefit criterion
	Personalized customization	5	5	4.4	Benefit criterion
	Cultural resonance	5.9	5	3.8	Benefit criterion
Experiential Requirements	Entertainment experience system	5	4.7	4.9	Benefit criterion
	Multi-scene compatibility	6.9	4.7	5.3	Benefit criterion
	Multi-sensory interaction	6.3	4.4	4.8	Benefit criterion
	Intelligent interaction	6.4	4.8	4.2	Benefit criterion

The values of $S_{i}$, $R_{i}$ and $Q_{\text{i}}$ are calculated according to (13), (14) and (15). The results of alternative evaluation are shown in Table 13.

**Table 13:** evaluation results of alternative solutions.

Alternatives	Group Utility $S_{i}$	Individual Regret $R_{i}$	Compromise Solution $Q_{i}$	Rank
Plan A	0.0526	0.0526	0	1
Plan B	0.6899	0.0526	0.4015	2
Plan C	0.8462	0.0526	0.5	3

According to calculations, the difference between Plan A and Plan B does not meet Condition 1. However, Plan A has the smallest $S_{i}$ and $R_{i}$ values and performs best in all criteria. Therefore, Plan A has high ranking stability and can be accepted without additional conditions.

#### 4.2.4. Stable diffusion controls the generation of the final solution.

**Fig 12:**
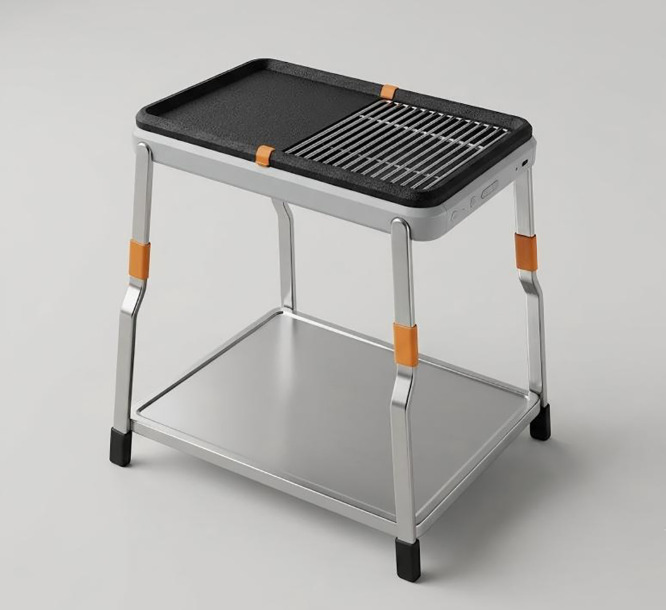
Visualization of Plan A.

Import Plan A into Stable Diffusion and generate a visual plan through the control network. The final result is shown in Fig 12. While maintaining accuracy, the product has a certain visual appeal, providing a conceptual prototype for subsequent product design and production.

#### 4.2.5. FCE of the final solution.

The FCE method was used to assess user satisfaction with the final plan. The assessment indicators were mainly based on five demand indicators in the camping cookware framework. It should be noted that the FCE evaluates user satisfaction across the five user-requirement dimensions and is not intended as an assessment of life-cycle sustainability. A total of 128 valid questionnaires were collected online and offline. The questionnaire had four evaluation indicators: dissatisfied, average, satisfied, and very satisfied. Participants made their selections based on the actual situation. The results are summarized in Table 14, which also includes the membership degree normalization calculated from each indicator.

**Table 14:** Final composite score.

Indicator	Dissatisfied	Average	Satisfied	Very Satisfied
Functional requirements	0	8	81	39
Safety requirements	1	10	69	48
Collaborative requirements	0	13	86	29
Emotional requirements	2	12	61	53
Experiential requirements	1	12	80	35
Membership degree normalization	0.0063	0.0859	0.5891	0.3188

The indicators were assigned values of 25, 50, 75, and 100, from low to high. The composite score of the camping cookware program was calculated to be 80. 508, corresponding to a satisfactory evaluation indicator, which verified the rationality of the program and indirectly verified the rationality of the human-AIGC collaborative process.

### 4.3. AIGC-assisted sustainable product design process

In the current context of global environmental degradation and depletion of natural resources, sustainable design is increasingly gaining prominence in product design [46]. Research has shown that approximately 80% of a product’s sustainability impact is determined during the design phase [47]. Additionally, the emergence of AIGC technology has opened up new possibilities for sustainable innovation in product design [48].

This study proposes an AIGC-assisted product design workflow that integrates user requirements, demonstrated through camping cookware concept design. The approach incorporates prompt formula cards, fuzzy generation with MidJourney, and controlled generation using Stable Diffusion with Control Net. The integration of AIGC enhances the sustainability of design output in multiple ways. The use of prompt formula cards enables the structuring and reuse of design knowledge, thereby reducing the time and resource costs associated with repetitive trial-and-error. MidJourney’s fuzzy generation supports rapid exploration of diverse design directions within a virtual environment, decreasing reliance on physical prototyping and minimizing material waste. Meanwhile, the Control Net enables precise, controlled generation, improving the accuracy and efficiency of design iterations while reducing unnecessary revisions. Overall, these methods promote sustainability by replacing physical processes with digital alternatives and improving workflow efficiency, thereby reducing resource use and environmental impact.

However, this study is limited to the conceptual design stage, so any material- and energy-related benefits should be regarded as potential rather than realized. Achieving full lifecycle sustainability would require further attention to production, distribution, and iteration, where reduced material use, lower energy consumption, and improved resource efficiency might be pursued but remain to be empirically verified. On this basis, exploratory directions are proposed to investigate how product innovation could align with sustainability objectives, with their actual material and energy gains yet to be validated in practice.

#### 4.3.1. Sustainable design principles and methods.

For the camping cookware design studied in this paper, there are a variety of sustainable design principles that can be followed. The 3R principle was the core framework for sustainable design in its early stages. With the continuous deepening of sustainable concepts, more extended principles have been derived, extending from the original 3R principle to 4R, 5R, and now 7R. The newly added Rs supplement and improve the practical path of sustainable design from different dimensions [49]. The 7R principle provides a more thorough and systematic approach to guiding the environmental sustainability of product and process design, promoting the harmonious development of economic, social, and environmental goals. refuse, and redesign, offering a more comprehensive and systematic approach to guiding the environmental sustainability of product and process design, thereby promoting the harmonious development of economic, social, and environmental objectives. Tracy Bhamra’s Design for Sustainable Behavior is a theoretical framework that integrates design theory, behavioral science, and systems thinking, aiming to influence user behavior through design interventions and promote the practice of sustainable lifestyles [50]. This concept breaks through the limitations of traditional product optimization, positioning design as a core tool for guiding behavioral change. Additionally, principles such as the life cycle principle and the Social Equity Design principle are not isolated but complementary and mutually reinforcing, collectively expanding the boundaries of sustainable design. This shift moves sustainable design from a single-dimensional approach toward a more comprehensive, holistic, and systematic direction, driving its continuous evolution to address the increasingly complex global challenges of sustainable development.

#### 4.3.2. Sustainable innovation design process for camping cookware.

**Fig 13:**
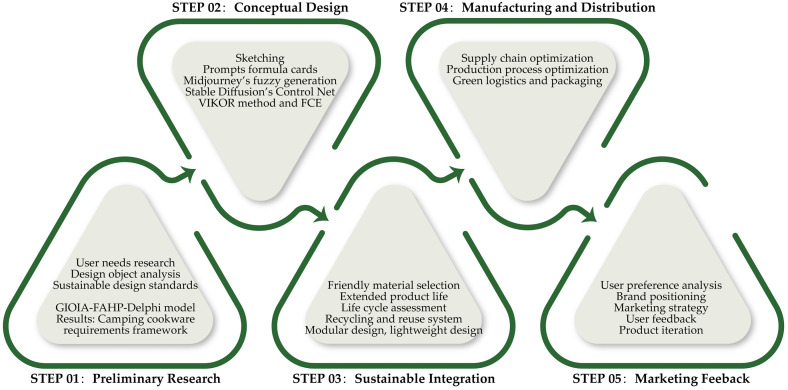
Product sustainable innovation design process.

The sustainable innovation design of camping cookware integrates sustainable concepts and AIGC technology. The innovation process consists of five stages: preliminary research stage, concept design stage, manufacturing and distribution stage, and marketing feedback stage. Adhering to a user-centered design philosophy, each stage considers sustainable concepts and explores a sustainable innovation design process that combines human and AIGC technology. The specific process is shown in Fig 13.

The workflow proceeds through five stages. First, the preliminary research phase analyzes user needs and design objects with emphasis on sustainability and existing applications, using GIOIA, FAHP, and Delphi to build a rigorous requirement framework for camping cookware that supports subsequent design. Second, in the conceptual design phase, Human–AIGC collaboration enables creative divergence and convergence: the framework constrains AIGC inputs, prompt-formula cards standardize wording, Midjourney fuzzy generation mitigates design fixation, VIKOR selects the preferred scheme, Stable Diffusion and Control Net refine visual quality, and FCE evaluates rationality. Third, sustainable design integration is central. Materials prioritize renewable and recyclable options to limit resource depletion and environmental impacts. Reduction and modularity extend service life and maintain compatibility with other kitchen equipment. Smart manufacturing and production optimization improve material efficiency and reduce emissions. Life-cycle assessment quantifies impacts across sourcing, manufacture, use, and end-of-life, while recycling and remanufacturing systems—supported by collection networks, online platforms, consumer education, and industry standards—enable recovery and reuse. Fourth, manufacturing and distribution integrate AI-driven design and production optimization with low-carbon logistics, including biodegradable packaging, route optimization, and the use of electric vehicles or National VI–compliant transport, which lowers the footprint and improves economics. Finally, marketing and user feedback focus on the mid- to high-end glamping segment, where research on target users and trends informs online and offline promotion, post-purchase feedback drives iteration, and incentive mechanisms encourage component replacement, disassembly, and reuse while protecting consumer rights.

## 5. Results and discussion

### 5.1. Constructing a framework for camping cookware requirements using the GIOIA method and the FAHP-delphi hybrid model

This study developed a requirements framework for camping cookware by collecting user data through interviews and the GIOIA method, with Maslow’s hierarchy of needs as the theoretical foundation. The framework elements were validated through multiple rounds of the Delphi method. The fuzzy analytic hierarchy process was then applied to assign weights to each element. The proposed cookware design was evaluated using fuzzy comprehensive evaluation and achieved an overall rating of satisfactory.

The framework comprehensively captures user demand characteristics for camping cookware across five levels. The results indicate that functional and safety requirements are the primary considerations, particularly usability and ergonomic adaptability. These are followed by experience related requirements, with increasing emphasis on intelligent interaction. Functions such as voice control, intelligent temperature regulation, and remote management can significantly improve both efficiency and user experience. Safety aspects must also be carefully addressed, including structural stability, safe materials, and integrated detection and alarm systems, which are essential in practical applications. In addition, the integration of social features enhances user engagement and strengthens emotional connections through collaborative use and group adaptability. The proposed framework fills a gap in existing research on camping cookware demand. In contrast, emotional factors, such as cultural resonance, personalized customization, and aesthetic appeal, are of relatively lower importance, with greater emphasis placed on functionality, safety, and user experience. Overall, the framework provides valuable support for designers and researchers in the early stages of product development.

The framework also improves AIGC input control by restructuring prompt combinations based on key elements and their assigned weights. This approach enhances output accuracy, increases experimental efficiency, and reduces resource consumption, thereby aligning with principles of sustainable design.

### 5.2. Using AIGC technology to build a camping cookware design process

This study developed an AIGC based design process for camping cookware by integrating prompt cards, Midjourney based fuzzy generation, and Control Net. Prompt cards improve the accuracy of AIGC inputs, while Midjourney reduces design fixation during idea generation. Stable Diffusion Control Net enables precise reconstruction of sketches, thereby enhancing the quality of design presentation. This workflow ensures accurate representation of user requirements and significantly improves design efficiency, thereby supporting product innovation.

### 5.3. Conceptualization of a sustainable product design platform with full-process AIGC integration

**Fig 14:**
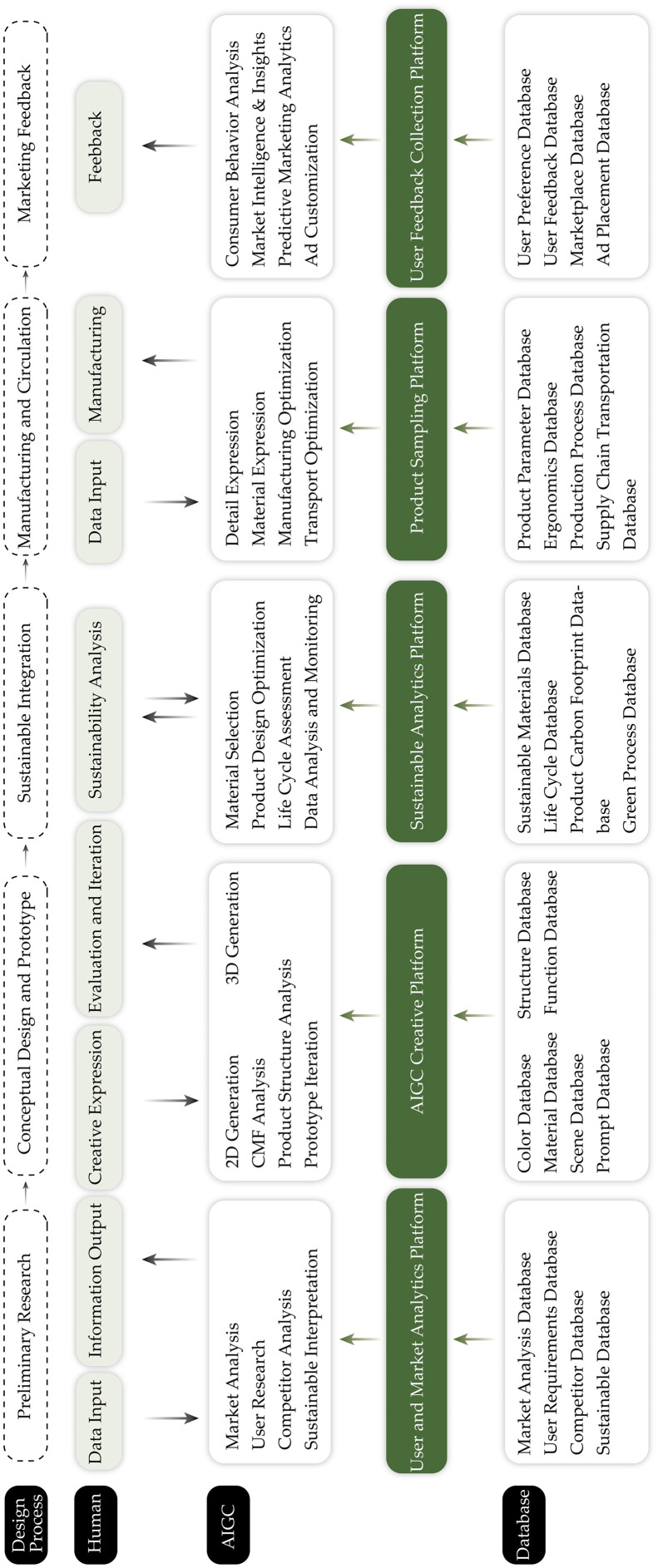
AIGC-driven sustainable product design platform.

Furthermore, this study advances a systematic AIGC-driven sustainable product design platform that extends beyond the conceptual phase to cover the entire design lifecycle, including preliminary research, concept development, sustainable design integration, manufacturing and distribution, and marketing feedback. As shown in Fig 14, this process is conceptually envisioned as a future-oriented platform framework entirely driven by AIGC. Unlike the five-stage practical workflow in Fig 13, which is based on a camping cookware case and focuses on current feasibility, this concept is more forward-looking and systematic, emphasizing deep empowerment and closed-loop driving by AIGC throughout the entire innovation process. The platform consists of an artificial intelligence service sub-platform and a supporting database, enabling seamless collaboration among designers, engineers, manufacturers, and consumers. In the preliminary research phase, AI-powered user and market analytics support demand forecasting, competitive analysis, and sustainable trend identification. During conceptual design, AIGC prioritizes sustainable elements such as material recyclability and weight reduction while supporting rendering, CMF selection, scenario adaptation, and digital twin generation. In the sustainable integration and life cycle assessment phases, the system evaluates low-carbon material combinations and generates carbon footprint reports. During manufacturing and distribution, AIGC optimizes green supply chains, logistics routes, and energy-efficient transportation. Finally, in the marketing feedback phase, AIGC facilitates advertisement generation, collects post-sale user feedback, establishes recycling channels, and feeds data back into the demand platform, forming a closed-loop sustainable design process. Overall, this study demonstrates how AIGC enables continuous, data-driven engagement throughout the entire design workflow, enhancing efficiency, reducing resource consumption, and promoting intelligent and sustainable product development.

### 5.4. Research limitations

This study has several limitations. First, although data were collected across multiple shopping platforms, the interview and expert participants were mainly Chinese. Differences in diet and lifestyle across cultures may limit how well the proposed camping cookware framework generalizes, so future work should include more diverse user groups to improve its cross-cultural applicability. Second, although fuzzy generation methods were used to reduce design fixation, whether Midjourney actually enhances creativity has not been empirically tested; comparative studies against conventional methods are needed to confirm this. Third, the study stops at the conceptual design stage and provides no concrete, quantifiable sustainability metrics, such as material reduction, energy savings from digital prototyping, or comparative efficiency gains. Its sustainability benefits should therefore be treated as potential rather than proven, and future work should add quantitative, life-cycle-based assessment to verify them.

## 6. Conclusions

This study identifies users’ practical needs for camping cookware in the context of glamping and proposes an innovative concept design. Using this case, an AIGC-assisted product concept design workflow is developed. The GIOIA method combined with FAHP–Delphi is applied to construct a requirement framework, in which functionality, safety, and user experience are prioritized, with ergonomics, usability, and intelligent interaction assigned the highest weights. Based on this framework, a design process incorporating prompt cards, MidJourney, and Stable Diffusion is implemented. The resulting cookware concept achieves a satisfactory fuzzy comprehensive evaluation score, demonstrating the feasibility of the proposed approach.

In addition, based on the case study, a sustainable product design framework is proposed that extends beyond the conceptual stage to encompass the full design process, including sustainable integration, production, and iterative feedback phases. This framework provides a theoretical reference for future product design research and promotes an approach that integrates technological development with broader sustainability considerations.

## Supporting information

S1 File In-depth interview outline. (DOCX)

S2 File Triangular fuzzy numbers information. (DOCX)
