# Supplementary material for: Redefining the design innovation process: Embedding sustainability through AI-generated camping cookware
Source: PLoS One. 2026 Aug 17;21(8):e0355687. doi: 10.1371/journal.pone.0355687 (PMC13480650; doi:10.1371/journal.pone.0355687)
Supplement: Supplementary file 1 [file pone.0355687.s001.docx]

**S1. In-depth interview outline**

| **Number** | **Question** |
| --- | --- |
| 1 | What's your first reaction when you see these outdoor cookware products？ |
| 2 | Could you choose your favorite product and share the reasons and your feelings？ |
| 3 | Which factors among these products do you think are not good and need improvement? You can offer your valuable suggestions？ |
| 4 | Do you have any considerations regarding sustainability for outdoor cookware products？ |
| 5 | What is your ideal outdoor cookware like and can it be described from any Angle？ |
