# Supplementary material for: Redefining the design innovation process: Embedding sustainability through AI-generated camping cookware
Source: PLoS One. 2026 Aug 17;21(8):e0355687. doi: 10.1371/journal.pone.0355687 (PMC13480650; doi:10.1371/journal.pone.0355687)
Supplement: Supplementary file 2 [file pone.0355687.s002.docx]

**S2. Triangular Fuzzy Numbers Information**

**The Meaning of The Median Value in Triangular Fuzzy Numbers**

| **Median of a Triangular Fuzzy Number** | **Meaning** |
| --- | --- |
| 1 | The two indicators are equally important |
| 3 | It means that the former is slightly more important than the latter |
| 5 | It means that the former is significantly more important than the latter |
| 7 | It means that the former is strongly more important than the latter |
| 9 | It means that the former is extremely more important than the latter |
| 2,4,6,8 | Represents the median value of the above judgment |

**Upper and Lower Bounds of Triangular Fuzzy Numbers**

| **Score Confidence** | **Stats** | **Digital Characteristics** | **Score Means** |
| --- | --- | --- | --- |
| High | 1 | (max(m-1/2,1), m, min(m+1/2,9)) | The scores given by experts are not ambiguous |
| low | 2 | (max(m-1,1), m, min(m+1,9)) | The scores given by experts are rather vague |
| average | 3 | (max(m-3/2,1), m, min(m+32,9)) | The scores given by experts are very vague |
